# Why are you looking at me? It’s because I’m talking, but mostly because I’m staring or not doing much

**DOI:** 10.3758/s13414-018-1588-6

**Published:** 2018-10-23

**Authors:** Hannah Scott, Jonathan P. Batten, Gustav Kuhn

**Affiliations:** 1 Department of Psychology, Goldsmiths, University of London, New Cross, London, SE14 6NW UK; 2 Department of Psychological Sciences, Birkbeck, University of London, London, UK

**Keywords:** Attention, Eye movements, Social cognition, Social attention, Audio visual interaction, Speech perception, Direct eye gaze

## Abstract

Our attention is particularly driven toward faces, especially the eyes, and there is much debate over the factors that modulate this social attentional orienting. Most of the previous research has presented faces in isolation, and we tried to address this shortcoming by measuring people’s eye movements whilst they observe more naturalistic and varied social interactions. Participants’ eye movements were monitored whilst they watched three different types of social interactions (monologue, manual activity, active attentional misdirection), which were either accompanied by the corresponding audio as speech or by silence. Our results showed that (1) participants spent more time looking at the face when the person was giving a monologue, than when he/she was carrying out manual activities, and in the latter case they spent more time fixating on the person’s hands. (2) Hearing speech significantly increases the amount of time participants spent looking at the face (this effect was relatively small), although this was not accounted for by any increase in mouth-oriented gaze. (3) Participants spent significantly more time fixating on the face when direct eye contact was established, and this drive to establish eye contact was significantly stronger in the manual activities than during the monologue. These results highlight people’s strategic top-down control over when they attend to faces and the eyes, and support the view that we use our eyes to signal non-verbal information.

**Electronic supplementary material:**

The online version of this article (10.3758/s13414-018-1588-6) contains supplementary material, which is available to authorized users.

## Introduction

We spend much of our time communicating with others, and successful social interactions require us to monitor other people’s intentions and desires. Although we rarely think about where to look, we systematically move our eyes to fixate on aspects of our environment that provide useful information. This attentional selection process is particularly important during social interactions in which faces, and in particular the eyes, provide lots of useful non-verbal information about a person’s mental state (Lansing & McConkie, 2003). Therefore, understanding the way in which we process these non-verbal cues is of great value.

Since the early works of Buswell (1935) and Yarbus (1967), it has been apparent that when we view pictures that contain people, we spend much of our time looking at faces (see also Birmingham, Bischof, & Kingstone, 2008a; Birmingham, Bischof, & Kingstone, 2008b, 2009a; Itier, Villate, & Ryan, 2007), which supports the view that our attentional systems are particularly attuned towards extracting socially relevant information. Moreover, people pay particularly close attention to other people’s eyes due to the amount of information that they can provide (Argyle & Cook, 1976; Birmingham, Bischof, & Kingstone, 2009b; Itier et al., 2007; Janik, Wellens, Goldberg, & Dellosso, 1978; Levy, Foulsham, & Kingstone, 2013). For example, another person’s gaze direction informs us about their intentions, as our attention is strongly influenced by directional eye gaze. Several studies have illustrated that people will automatically follow a person’s gaze (Kuhn & Kingstone, 2009; Ricciardelli, Bricolo, Aglioti, & Chelazzi, 2002), and that we are particularly attracted to eyes that look directly at us to establish direct eye contact (Kleinke, 1986). For example, new-borns look at faces displaying direct gaze for longer than when the gaze is averted, suggesting that this sensitivity towards direct eye gaze develops early on (Farroni, Csibra, Simion, & Johnson, 2002). As adults, we detect faces with direct eye gaze more rapidly (vonGrunau & Anston, 1995), and this direct eye contact is also more effective at holding our attention (Senju & Hasegawa, 2005). This has led to the suggestion that direct eye gaze automatically captures our attention.

The eyes are certainly of great social importance, but there are numerous contexts in which other parts of our face provide useful information. For example, talking lip movements provide a great deal of information about the words being spoken (J. Macdonald & McGurk, 1978). Several other studies have shown that when participants focus on the content of speech, they spend more time looking at the mouth than the eyes, presumably to aid the understanding of speech (Buchan, Pare, & Munhall, 2007; Lansing & McConkie, 2003; Yehia, Rubin, & Vatikiotis-Bateson, 1998). Lansing and McConkie (2003) found that people tended to fixate on the eyes, but fixated more on the mouth when speech was inaudible, with further research reporting that fixations to the mouth were particularly apparent when a speech perception task was made more difficult by adding ambient noise to the videos (Buchan et al., 2007). The authors suggested that viewers attempt to compensate for the poor auditory information by lip-reading, thus focusing more on the mouth than the eyes. However, Foulsham and Sanderson (2013) monitored participants’ eye movements whilst they watched people having a discussion, with half of the clips being shown with sound and half without. Whilst the sound significantly influenced which of the people in the group were fixated on, the sound did not influence the amount of time they spent looking at the eyes compared to the mouth, suggesting that people do not necessarily look at the mouth to compensate for the lack of sound input. Võ, Smith, Mital, and Henderson’s (2012) research showed that the removal of audio information in fact can result in a decrease in the number of fixations to the face when viewing clips of people talking to a camera. Fixations on the mouth are particularly decreased, and fixations on the background of the video increase. The authors suggested that the lack of sound reduced participants’ interest in the video and thus resulted in less attentional focus.

All of the research reviewed so far has used video stimuli in which participants watch people who are simply talking to the camera, or holding a relatively static conversation, and few have involved situations that include or involve hand movements. So, whilst the face provides many useful clues about social information, other aspects of our body also convey important information. A developmental study by Fausey, Jayaraman, and Smith (2016) described an early transition in infant gaze behaviour from mostly fixating faces to increasingly fixating the hands during the first 2 years, which is evidence of the emergence of object relationship and hand cue understanding. We often supplement our verbal language with gestures that are used to resolve ambiguities and thus improve communication (Krauss, Chen, & Chawla, 1996). Similarly, pointing gestures are often used to direct (Langton & Bruce, 2000) or misdirect where we look (Kuhn & Tatler, 2005; Otero-Millan, Macknik, Robbins, & Martinez-Conde, 2011). For example, much of the research on attentional misdirection relies on clips of magicians using misdirection to carry out magic tricks and illustrates that whilst social cues (i.e., where the magician is looking) are important in directing attention, people spend much of their time looking at the magician’s hands (Kuhn & Land, 2006; Kuhn & Tatler, 2005; Kuhn, Tatler, & Cole, 2009; Kuhn, Teszka, Tenaw, & Kingstone, 2016). People also often watch their own hands whilst carrying out goal-directed movements, to ensure they are doing the task correctly (Neggers & Bekkering, 2000). Moreover, Flanagan and Johansson (2003) showed that participants employed a predictive gaze pattern when watching manual actions; thus participants primarily engaged in predicting the next stage of the task being carried out. Another person’s hand movements provide knowledge about what they are doing, and so at times the hands may convey more important information than the face. However, little is known about how these hand movements influence our attentional process and in particular how this may interact with hearing speech.

Our primary aim was to investigate whether hearing corresponding speech would drive the observers’ eyes to the face, and whether these findings generalize to situations in which people use hand movements to communicate, either by demonstrating a manual activity or by purposefully misdirecting the observer’s attention. There is a general consensus in the literature that we prioritize faces, but faces may become less important when other cues, such as gestures and manual actions, are available. We predicted that when people watch an individual performing a manual task it would reduce the amount of time they spend looking at the face and they would focus on the person’s hands instead. We created three different contexts that varied in the extent to which hands and gestures were used to direct attention. In the monologue condition, a person simply spoke to the camera; although the hands are used for simple gestures, they do not manipulate objects. In the second condition the person talked about a manual activity (making a cup of tea), and the hands conveyed important information as they were used to demonstrate the activity. In the final condition, the hands were used to actively misdirect attention. Here, a magician performed a magic routine in which both speech and hand movements were used to actively misdirect the viewer’s attention.

Our second objective was to investigate whether the presence of audible speech will bias people to look at the speaker’s face, and in particular towards the mouth. Based on previous research we predicted that people would spend more time looking at faces, particularly the mouth, if they could hear the interaction compared to viewing the activity with no sound. When the hands are used to convey additional information, social cues derived from the face are likely to be of less importance. We therefore predict that the effect of speech would be most apparent during a monologue where very little other information could be gained from hand movement.

Finally, we explored the role of direct eye contact in attracting attention. Past research suggests that direct eye gaze will automatically capture people’s attention (Senju & Hasegawa, 2005), which implies that people should look at the face whenever eye contact is established. If direct eye gaze automatically captures people’s attention over other salient aspects of a scene, we would expect people to spend more time fixating on faces when eye contact is established, an effect that should be independent of what the person is doing. However, more recently it has been suggested that within a social context we predominantly use our eyes as non-verbal signals (Gobel, Kim, & Richardson, 2015; Kuhn et al., 2016; Wu, Bischof, & Kingstone, 2013), which implies that we have more control over where we look, and thus fixate on the face when there is a communicative benefit as opposed to simply when automatically drawn to it. We therefore predict that direct eye gaze will draw attention more strongly towards the person’s face when the eye contact is directly used to communicate with the observer (i.e., manual action and misdirection) than when the person is simply holding a monologue.

## Method

### Participants

Seventy-two Psychology Undergraduate students participated for course credits (46 female; 26 male; mean age = 22.0 years, SD = 4.67) and all had normal or corrected-to-normal vision. Random selection was used to assign half of the participants to the sound condition, and half to the silent condition. They were then further divided into one of six sub-groups to control for the order in which the three videos were presented.

### Material

**Fig. 1:**
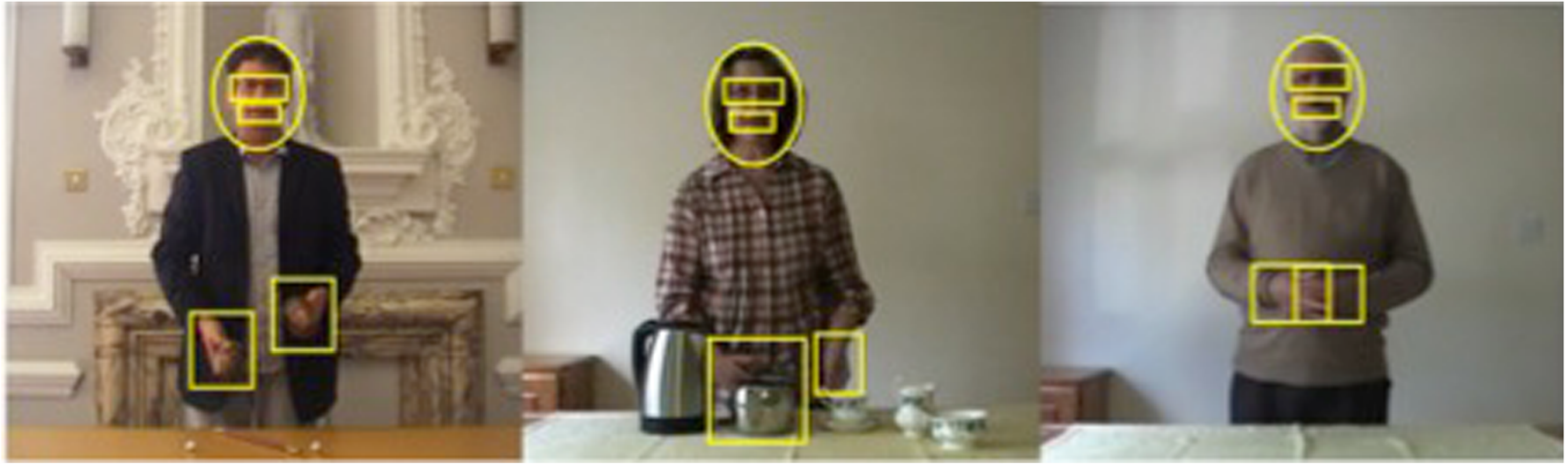
Stills of the Misdirection, Manual action and Monologue videos (left to right) with interest areas outlined. The face interest area consisted of an oval shape that covered the entire face. Two sub-interest areas were created which covered the eyes and the mouth. The hands interest area covered both of the actor’s hands, as well as the objects that were touched in each particular frame, and the size of the interest area was expanded so that it covered these objects

Three different actors were recorded carrying out one of the three tasks. The three video clips were recorded in standard definition (720 × 576 pixels) in mp4 format and the camera was positioned on a tripod 2.3 m away from the actor (1.3 m high). This ensured that all of the videos were the same size relative to the screen (see Fig. 1). The monologue consisted of a man talking about the River Shannon (186 s). The manual action involved a woman demonstrating how to make a perfect cup of tea (136 s). The misdirection condition involved a male magician performing a traditional demonstration of the cups and balls trick (151 s). All videos were shot against a static relatively plain background to prevent environmental factors from drawing attention away from the actor. The actors in each video all stood behind a waist-high table, so their upper bodies were entirely visible (see Fig. 1).

Eye movements were recorded using an Eyelink 1000 eye tracker (SR-Research), which uses a remote camera and a head support (viewing distance = 57 cm). Eye movements were recoded monocularly at 500 Hz. The videos (30 frames/s) were presented on a 22-in. ViewSonic Monitor using Experiment Builder (SR-Research) presentation software, which ensured accurate frame display timing. The monitor was run at 60 Hz and the resolution was set to 800 × 600, which meant the video filled most of the visible area. The audio was presented using Sennheiser headphones.

### Procedure

Participants were randomly assigned to the sound or no-sound condition, and told that they would view three short video clips whilst having their eye movements monitored. Participants were told they were not expected to carry out any specific tasks and were encouraged to relax whilst they watched the videos. Participants in the silent condition were warned that the video would have no sound.

Eye movements were calibrated using a 9-point calibration and validation procedure, and each trial started with a central fixation point, which acted as a further calibration check. For each of the video clips we also measured levels of engagement using items taken from Webster et al. (1993). This scale was designed to measure engagement in human-computer interactions and a total of six questions from the “Attention Focus” and the “Intrinsic Interest” category were used (distraction, absorption, boredom, interest, fun, attentional focus).

### Data preparation

All of the eye movement analysis was carried out using Data Viewer version 2.3.0.73 (SR-Research). Since all aspects of the video we were interested in moved across time, we manually coded dynamic interest areas on a frame-by-frame basis. The interest areas were locked to the target and changed location and size as the target moved (see Supplementary Videos). The face interest area consisted of an oval shape that covered the entire face. Two sub-interest areas were created that covered the eyes and the mouth. The second main interest area was the hands, which covered both of the actor’s hands as well as the objects that were being used in that particular frame, with the size of the interest area adjusted so that it also covered this object. Table 1 shows the mean sizes of the different interest areas of each of the videos. As the videos were of different duration, we only analysed the data from the first 136 s, thus ensuring that they were matched in length.

**Table 1:** Mean sizes (% of the surface area) of interest areas for each video. Please note that the interest area sizes varied across time. The eyes and mouth interest areas were subsets of the face area

Activity	Interest area size (%)			
	Face	Eyes	Mouth	Hands
Monologue	3.64	0.26	0.15	2.61
Manual action	6.84	0.44	0.2	7.29
Misdirection	4.06	0.38	0.17	3.99

## Results

Three main analyses were carried out that were designed to best answer the research questions and understand the differences in time spent looking at the different interest areas depending on the sound condition, viewed activity, and during periods with direct gaze. Preliminary analysis of the data showed that gender did not have a significant impact on the data, and so it was not included in the main analyses as a covariate.

### Effect of activity and sound on fixating on the face and hands

**Fig. 2:**
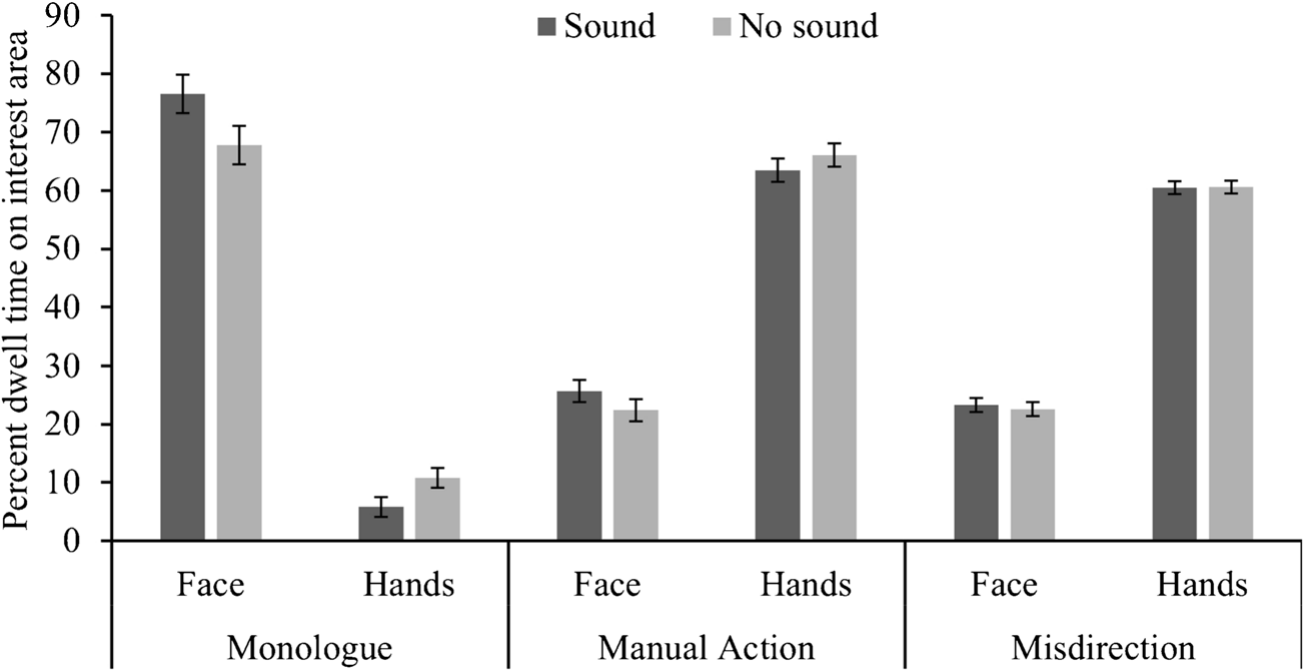
Mean percent dwell times for fixating the face and hands as a function of video and sound condition (error bars denote ±1 standard error)

The first analysis looked at whether the sound and the activity influenced the degree to which participants fixated on the face as opposed to the hands. We calculated the percentage of time that participants fixated each interest area, and carried out an ANOVA with activity (monologue, manual action misdirection) and interest area (face, hands) as the within-participant variables, and sound (sound, no sound) as the between-participants variable. Figure 2 shows the mean dwell times for each of these interest areas and videos as a function of sound condition.

Our primary interest was whether the sound condition oriented participants’ eye movements to the actor’s face; our results did indeed reveal a significant audio by interest area interaction, *F*(1, 70) = 4.30, *p* = .042, η^2^= .11. The interaction was broken down by looking at the simple effects. As predicted, participants spent significantly more time fixating on the face with sound rather than without sound [*t*(70) = 1.87, *p* = .033 (one-tailed), *d* = 0.31], and vice versa for the hands [*t*(70) = 1.68, *p* = .048 (one-tailed), *d*= 0.28]. Whilst we feel justified in using one-tailed tests to assess the simple effect, we would like to draw attention to the fact that these effects are rather small and are no longer significant under stricter criteria. There was no significant activity by sound by interest area interaction *F*(2, 140) = 0.708, *p* = .50, η^2^= .01, which suggests this effect was independent of activity.

Our secondary interest was whether the actor’s activity influenced the duration to which participants fixated the face as opposed to the hands. There was a significant activity by interest area interaction *F*(2,140) = 652, *p* < .001, η^2^= .90, and Bonferroni corrected t-tests were applied to break down the effect. Participants spent significantly more time fixating on the face than the hands in the monologue video [*t*(71) = 19.3, *p* < .001, *d* = 2.28], but the opposite pattern was found in both the manual action [*t*(71) = 15.9, p < .001, *d* = 1.87] and misdirection [*t*(71) = 26.3, p < .001, *d* = 3.10]. As predicted the participants spent significantly more time looking at the hands than the face in the two videos that included informative hand movements. The ANOVA also revealed a significant main effects of activity, *F*(2, 140) = 16.0, *p* < .001, η^2^= .19, and interest area *F*(1, 70) = 8.67, *p* = .004, η^2^= .11. The main effect of sound was not significant, *F*(1, 34) = 0.67, *p* = .41, η^2^= .009, neither was the interaction between sound and activity, *F*(2, 68) = 0.71, *p* = .50, η^2^= .01. None of these main effects or interactions are particularly meaningful in light of the predictions.^1^

### Effect of task and sound on fixation to the eyes and mouth

**Fig. 3:**
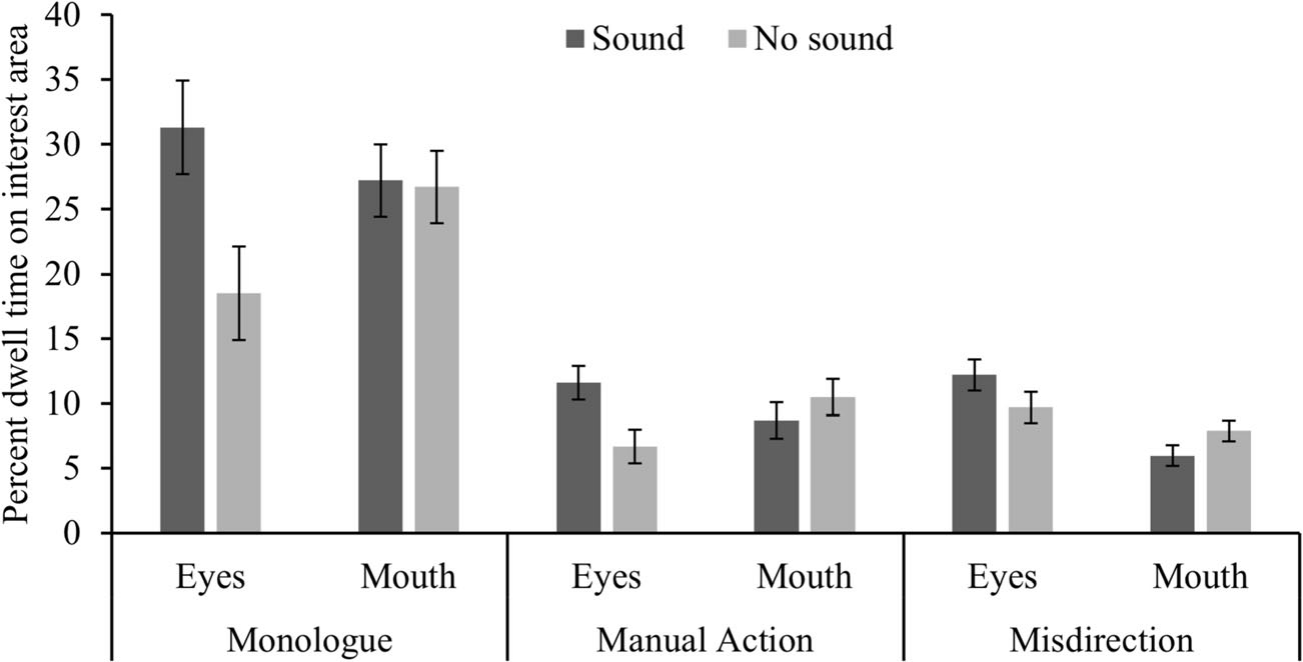
Mean percent dwell times for fixating the eyes and mouth as a function of activity and sound condition (error bars denote ±1 standard error). These interest areas are subsets of the earlier analysed face interest area

The second analysis addressed whether hearing sound (mostly speech) as opposed to hearing no sound would draw people’s fixations towards the mouth rather than the eyes. For this analysis only data that fell within the eye or mouth interest areas were considered. It is clear from comparing Figs. 2 and 3, that roughly 75%^2^ of the fixations on the face are captured by the eyes and mouth. A further factorial ANOVA contrasted gaze durations to the two interest areas (eyes and mouth) by activity (monologue, manual action, misdirection) and sound condition (sound or no sound). There was a significant interest area by sound interaction, *F*(2, 140) = 3.99, *p* = .05, η^2^= .054. Contrary to our prediction (that sound would increase gaze to the mouth), Bonferroni corrected post-hoc comparisons showed that participants spent significantly more time fixating the eyes when there was sound than with no sound [*t*(70) = 2.70, *p* = .009, d = 0.64], but the difference between the sound conditions was not significant for the mouth *t*(70) = 0.52, *p* = .60, d = 0.13]. Hearing the corresponding sound influenced the amount of time that was spent fixating the eyes but not the mouth.

There was no significant interest area by sound by activity interaction *F*(2, 140) = 1.01, *p* = .37, η^2^= .014. There was a significant main effect of sound, *F*(1, 70) = 6.28, *p* = .015, η^2^= .082, but not a significant main effect of interest area *F*(1, 70) = 0.966, *p* = .80, η^2^= .001, or interest area by activity interaction *F*(2,140) = 2.42, *p* = .092 η^2^= .033. There was a significant sound by activity interaction *F*(2,140) = 6.33, *p* = .002, η^2^= .083, and a significant main effect of activity, *F*(2,140) = 215, *p* < .001, η^2^= .76, but no significant interest area by activity interaction *F*(2,140) = 2.42, *p* = .092, η^2^= .033.

### Effect of gaze direction fixations to the face

**Fig. 4:**
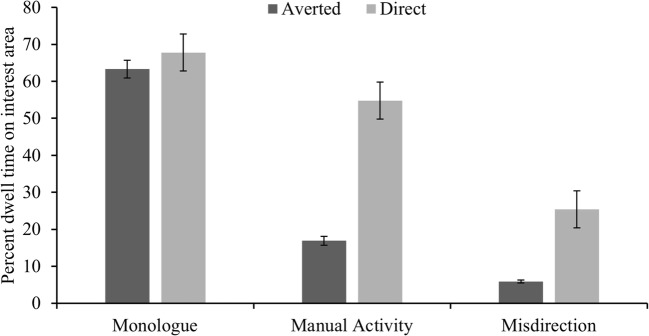
Mean percent dwell times for fixating the face as a function of whether the filmed person’s gaze was directed towards the observer (direct) or averted

Finally, we examined the extent to which establishing eye contact made people fixate on the face. For each video, we manually coded (frame by frame) whether the actor’s gaze was directed towards the camera (direct) or whether they were looking elsewhere (averted). In the monologue the actor maintained direct eye contact 48% of the time, compared to 15% in the manual action and 33% in the misdirection condition. Figure 4 shows the percentage of time spent fixating on the face as a function of activity and whether the actor maintained direct eye contact or when their gaze was averted. An ANOVA with activity (monologue, manual action, misdirection) and gaze direction (direct, averted), as a within-subjects factor found a significant main effect of activity, *F*(2, 140) = 287, *p* <.0005, η^2^ = .80, mirroring the findings reported in the earlier analysis (activities that required hand actions oriented gaze to the hands more). More importantly there was a significant main effect of gaze direction *F*(2, 140) = 795, *p* < .00005, η^2^ = .92 , and a significant gaze direction by activity interaction, *F*(2, 140) = 235, *p* < .00005, η^2^ = .77. The activity being viewed modulated the extent to which people’s eyes were drawn towards direct eye gaze. Although participants spent more time looking at the face when direct eye contact was established (all *p* <.0005), post-hoc t-tests (difference scores were calculated by subtracting the % dwell time when gaze was averted from when gaze was directed towards the observer) (Bonferroni corrected) revealed that the difference in fixations to the face as a function of gaze direction was significantly smaller in the monologue than in both the manual task [*t*(71) = 20.7, *p* <.0005, d = 2.44] and the misdirection condition [*t*(71) = 11.1, *p* <.0005, d = 1.31]. Moreover, the difference was significantly greater in the manual than in the misdirection task, *t*(71) = 11.4, *p* < .0005, d = 1.35. Thus, eye contact was significantly more effective at driving fixations to the face during the viewing of the manual activities than during the monologue. We also ran the same analysis including sound as a variable, but none of the interactions involving sound or the main effect of sound were significant (all p > .09).

### Engagement

Table 2 shows the engagement scores for each of the videos as a function of sound condition. Data from three participants are missing due to data loss. An ANOVA with activity as within subject factor and sound condition as between subject factor found a significant main effect of activity *F*(2, 135) = 50.2, *p* < .0005, η^2^= .43, but no significant main effect of sound *F*(1, 67) = 2.27, *p* = .12, η^2^= .038, and no significant sound by activity interaction *F*(2, 134) = 2.00, *p* = .14, η^2^= ,029. Whilst there were clear differences in engagement for the different videos, the sound did not significantly influence the participant’s reported level of engagement.

**Table 2:** Mean engagement scores for the three videos as a function of audio signal (standard errors in parenthesis)

	Activity		
	Monologue	Manual action	Misdirection
Sound	15.7 (0.713)	17.3 (0.685)	21.7 (0.741)
No sound	13.2 (0.682)	17.5 (0.655)	21.3 (0.710)

## Discussion

Our main objective was to explore the factors that drive people’s attention towards the face, specifically whether corresponding sound and the type of action shown altered viewing behaviour.

### Is there a general bias to look at faces?

Although participants spent a substantial proportion of their time fixating on the face, this bias was strongly influenced by what the actor was doing. When the actor simply held a monologue and did not do much else, participants spent around 70% of their time looking at the person’s face, and about 25% of the time was spent fixating on the eyes. Previous researchers have suggested that there is a general bias towards looking at faces, and in particular the eyes (Birmingham et al., 2009a, b; Emery, 2000; Walker-Smith, Gale, & Findlay, 1977). Our results only partially support this view. Most previous research has measured people’s eye movements whilst they viewed static images containing individuals, or scenarios that simply involve a person speaking to a camera without any other movement (e.g., Vo et al., 2012). In this latter study participants spent nearly 90% of their time fixating the face.^3^ Our results clearly illustrate that within a relatively passive context such as a monologue there is a strong bias towards fixating on faces, but this bias is strongly modulated by context. In the two conditions where the actor used their hands to do things (i.e., make a cup of tea or perform magic tricks), fixations were biased towards the hands, with fixations to the face dropping to nearly 20%. Previous research may have artificially inflated the extent to which we prioritize faces (particularly the eyes), simply because they measured eye movements during situations in which there was very little else happening. Unlike the monologue condition, the other video clips involved instructional hand movements, and it would be interesting for future research to investigate whether eye movements to the hands are driven by the hands’ instructional nature (i.e., high level) or simply due to low level motion signals.

### Does auditory information influence where we look?

Our second objective was to investigate whether the presence of the corresponding sound (mostly formed of speech), influenced people’s tendency to look at the face, and in particular the mouth (the dominant sound source). The results showed that the presence of sound had a significant yet relatively small effect on where people looked. Hearing the audio increased the amount of time participants spent fixating the face. Results from past research have been rather mixed, some having reported increased fixations to the face when audible speech is present (Foulsham & Sanderson, 2013), with others having reported a reduction in the presence of audible speech (Vo et al., 2012). Vo et al. (2012) showed that when the speech sound was removed, observers spent less time looking at the face and fixated aspects of the background instead. More recent studies have suggested that individual differences have an impact on any proposed systematic differences in gaze to the face, particularly in more real-world studies such as this (Peterson, Lin, Zaun, & Kanwisher, 2016). The authors suggested that the absence of the speech made the video clips less engaging and thus encouraged observers to look elsewhere. We directly measured participants’ engagement in the different videos and, rather surprisingly, the sound did not significantly influence people’s self-reported engagement in the clips. It is likely that our participants were sufficiently engaged in watching the videos even without sound.

Vo et al. (2012) reported that audible speech increased people’s fixations to the mouth from 23% to 31%. Our results do not support these findings. Hearing the speech (when the sound was present) resulted in significantly more time fixating on the eyes than when there was no sound, but had no significant effect on time spent fixating on the mouth. In many previous studies (e.g., Vo et al., 2012; Foulsham & Sanderson, 2013), faces filled most of the screen, whilst in this study the framing of the activity meant that the face was proportionately smaller on the screen. It is important to note that our eye tracker and our analysis tools were technologically advanced enough to distinguish between fixations in the different interest areas, and so it is unlikely that this difference was due to errors in the eye-tracking signal. Instead, these differences probably reflect genuine changes in eye-movement strategies that result from presenting different sized faces (i.e., different retinal image size). If we are presented with a large face, we have to be more strategic about where we look, as fixating on the eyes will make it more difficult to process information from the mouth or eyes if they are in the peripheral vision. However, if the presented face is small enough for most of it to be processed by the fovea (i.e., central vision), we have to be far less strategic as to where we look. This argument is supported by similar research that examines the use of close-up and far-away shots of people in cinematography; directors use close-up shots of faces to gain more control over where the viewer looks. Empirical evidence (Loschky, Larson, Magliano, & Smith, 2015) supports this idea by showing this method employs the viewers’ automatic attention over their understanding of the context of the shot. It seems that the inclusion of audible speech does not simply increase our tendency to look at faces, or the mouth in particular, and instead the mechanism responsible for this shift in attention is modulated by other factors such as task or size of the face.

### Does direct eye contact attract attention?

Our third objective was to investigate the extent to which direct eye contact attracts people’s attention. Much of the past research suggests that direct gaze will automatically capture our attention (Senju & Hasegawa, 2005; vonGrunau & Anston, 1995). Our results showed that observers were much more likely to fixate on the face when the actor looked towards the camera than when the gaze was averted. However, the filmed presenters’ gaze direction had a stronger influence on the time participants spent fixating the face in the manual action and the misdirection condition than in the monologue condition, which suggests that people have some strategic control over whether they attend to the direct gaze or not. Why may this be the case?

One potential explanation is that averted gaze makes the eyes seem less salient, and thus less attention grabbing. However, we propose that there is a qualitative difference in how gaze is used when explaining a manual task than when simply holding a monologue. During social interactions, or when explaining things using real objects, we use our gaze as a non-verbal signal (Gobel et al., 2015) to refer to objects (R. G. Macdonald & Tatler, 2013, 2015), and in our study, establishing gaze contact usually coincided with a pause in actions and generally signalled a point at which explanation about the actions were provided verbally. During the monologue, a person’s gaze was used far less strategically (as the presenter could not see or interact with the viewer), and thus did not provide much valuable information. Therefore, whilst establishing eye contact commonly results in people attending to the face, this is done so strategically and is more likely in situations when direct gaze conveys information.

These findings coincide with results from a recent study where we showed that establishing eye contact whilst asking a verbal question was extremely effective at drawing overt attention to the face, but similar to our results, observers did have some control over whether they did so or not (Kuhn et al., 2016). Another study by Freeth, Foulsham, and Kingstone (2013) noted that participants looked more at the experimenter’s face than other parts of the scene when direct eye contact was made. They found this to be the case only in live interaction, not video interaction, whilst other gaze patterns did not significantly differ between the measured video and live interaction, likely due to the artificiality of the interaction through video. It is unlikely that this tendency to fixate the face in response to direct gaze was related to speech processing, and so the effect was independent of whether the speech was audible or not. Instead, it is more likely that these eye movements to the face served as non-verbal signals, acknowledging that observers are following the action, which points to our general use of eye movements as a form of non-verbal signalling (Gobel et al., 2015; Kuhn et al., 2016; Wu et al., 2013; Wu, Bischof, & Kingstone, 2014).

### Limitations

Our results clearly illustrate that the presence of audible speech is dwarfed by our other two variables, namely whether the person is using his/her hands to demonstrate the actions, and whether direct eye contact is established or not. It is important to note that there were some limitations to this study. Whilst our results showed a minimal effect of audio, the speech content in each of the three videos was different, and the videos also contained different people, which potentially could elicit some differences in gaze patterns and fixations. Moreover, there were some variations in background between the three videos, though our analysis suggests that these minimal scene differences did not have a major impact on our results. Additionally, we have assumed here that direct gaze is what draws attention to the face. Given previously published literature this is the most likely explanation, but there may be other factors that draw individuals to the face in light of individual differences in fixation patterns that will require extensive further research to be fully understood. Moreover, since most of the manual actions occurred in the bottom half of the visual field, we cannot rule out the possibility that fixations to the hands were simply due to any moving stimulus presented in the lower visual field. However, other research has shown that people spend little time fixating gestures (Gullberg & Holmqvist, 2002), and that fixations on gestures are independent of the hands’ physical location (Gullberg & Kita, 2009). We are therefore fairly confident that the effects are due to the meaningful hand movements. Our results also showed that the filmed presenter’s gaze direction had a stronger influence on driving peoples gaze to the face in the manual action and the misdirection condition than in the monologue condition. Since participants spent significantly more time fixating the face in the latter condition, it is possible that ceiling effects contributed to these differences. However, is important to note that this effect was very reliable, and we therefore think it is unlikely that ceiling effects can account for the differences entirely.

### Conclusion

By studying attentional processes in relatively passive contexts we may have exaggerated the importance that faces play in attracting our attention. Our results clearly illustrate that when we use our hands to handle objects, or to misdirect attention during a magic trick, people spend more time watching the hands than the face. Our hands seem to play just as important a role in orienting people’s attention as our eyes do. Being able to hear a person’s voice did lead to more fixations towards the face, but this effect was much smaller than when the person established eye contact. Fixating on the face in response to direct gaze was much more pronounced in the manual activity than the monologue video clips, which suggests that people have a great amount of active top-down control over whether the face is fixated on or not. We hypothesize that these eye movements to the face in response to direct eye contact serve as non-verbal acknowledgments and form a crucial part of our non-verbal communication.

## Electronic supplementary material

ESM 1 (ZIP 62.4 mb)

## Footnotes

1 To ensure that the different sizes of interest areas had no significant impact on our results, extra analyses were conducted. All the dwell time values for each participant were standardized based on the percentage of space on the screen that each interest area occupied. Using these standardized measures, participants spent significantly more time fixating the face than the hands in the monologue condition [t(71) = 16.0, p <.0005], but significantly less time fixating the face than the hands in the manual action [t(71 = 15.9, p <.0005] and the misdirection conditions [t(71) = 26.8, p < .0005].

2 Calculated by dividing proportion dwell time spent on mouth and eyes, by dwell time on face as a whole.

3 It is important to note that in Vo et al. (2012) the face covered most of the screen, which may account for the larger amount of time spent fixating the face.
